# Nanoparticle Induced Cell Magneto-Rotation: Monitoring Morphology, Stress and Drug Sensitivity of a Suspended Single Cancer Cell

**DOI:** 10.1371/journal.pone.0028475

**Published:** 2011-12-13

**Authors:** Remy Elbez, Brandon H. McNaughton, Lalit Patel, Kenneth J. Pienta, Raoul Kopelman

**Affiliations:** 1 Department of Applied Physics, University of Michigan, Ann Arbor, Michigan, United States of America; 2 Department of Biomedical Engineering, University of Michigan, Ann Arbor, Michigan, United States of America; 3 Department of Internal Medicine, University of Michigan School of Medicine, Ann Arbor, Michigan, United States of America; 4 Department of Urology, University of Michigan School of Medicine, Ann Arbor, Michigan, United States of America; 5 Department of Chemistry, University of Michigan, Ann Arbor, Michigan, United States of America; Michigan State University, United States of America

## Abstract

Single cell analysis has allowed critical discoveries in drug testing, immunobiology and stem cell research. In addition, a change from two to three dimensional growth conditions radically affects cell behavior. This already resulted in new observations on gene expression and communication networks and in better predictions of cell responses to their environment. However, it is still difficult to study the size and shape of single cells that are freely suspended, where morphological changes are highly significant. Described here is a new method for quantitative real time monitoring of cell size and morphology, on single live suspended cancer cells, unconfined in three dimensions. The precision is comparable to that of the best optical microscopes, but, in contrast, there is no need for confining the cell to the imaging plane. The here first introduced *cell magnetorotation* (CM) method is made possible by *nanoparticle induced cell magnetization*. By using a rotating magnetic field, the magnetically labeled cell is actively rotated, and the rotational period is measured in real-time. A change in morphology induces a change in the rotational period of the suspended cell (e.g. when the cell gets bigger it rotates slower). The ability to monitor, in real time, cell swelling or death, at the single cell level, is demonstrated. This method could thus be used for multiplexed real time single cell morphology analysis, with implications for drug testing, drug discovery, genomics and three-dimensional culturing.

## Introduction

The heterogeneity, i.e. non-uniformity, found in cancer cell populations, and the ubiquitous cell differentiation, has led to increased interest in individual cell studies [1]–[4]. Historically, a tumor was thought to originate from the successive divisions of a single ‘mother cell’, leading to the assumption that all the cells in a tumor shared the same genetic code. However, recent findings have altered this theory, stressing the need for tools that can monitor and track single cells in a high throughput fashion [5]–[8]. Currently, standard assays performed on cell populations make individual patterns difficult to access, due to effects of averaging [9]. Flow cytometry, for instance, has been massively used in the last 20 years, for its ability to perform fast analysis on a very high number of cells at a time (10000 cells/s). Time point analysis can also be performed using this technique, but it is not possible to track each cell individually.

Then again, it is especially important that even a small minority of cells, such as stem cells, whose behavior could be considered to be statistically irrelevant compared to the large majority of the population, can have a critical biological and medical impact. For instance, the use of the *Imatinib* drug that targets the *BCR-abl* fusion protein in patients with chronic myelogenous leukemia (CML) first seemed to be one of the most successful targeted therapies. However, the treatment does not eliminate the CML stem cells, and with the withdrawal of Imatinib the disease reappeared [10], [11]. As a consequence, the focus on cell-to-cell variations has also allowed important breakthroughs in the understanding of cell differentiation, drug response, protein mechanisms and dynamics, as well as of the important role played by stem cells, especially for cancer stem cells [12]. Metastasis relies on cancer cells circulating in the vascular network. The cells responsible for cancer propagation to secondary tumor sites are extremely rare (a few cells per million in the blood), and they go through a circulating stage before populating other tissues. Therefore, along with single cell analysis, three dimensional assays also permit a better comprehension of cellular dynamics [13]–[15], by narrowing the gap between *in vitro* and *in vivo* behavior [7]. However, all previously mentioned single cell analysis techniques are restricted by their confinement of the cell in two dimensions. To overcome this limitation, we employ a new approach using *suspended cell magneto-rotation* (CM).

Specifically, we use a *nanoparticle induced Cell Magneto-rotation method*, where the driving magnetic field and the rotating cell are *out-of-synch* with each other. The cells are embedded with 30 nm commercial magnetic nanoparticles (Ocean Nanotech®) and are rotated under an external magnetic field of about 1 mT, at about100 Hz. We note that a thousand times (1000×) higher fields, on the order of 1T, are used for MRI. Also, magnetic nanoparticles have been widely used in biology [16]–[20]. Thus the CM method is designed to be biocompatible and non-toxic. The live cell is rotated *asynchronously* (see **Supplementary Information S1**) in suspension, and its rotational frequency is highly sensitive to any morphology change. As reported here, magneto-rotation does not affect the cell's viability, and allows for real time analysis to be performed. Changes in cell morphology are indicated quantitatively by the single cell's rotation period. The trends in the rotation rate allow discrimination between a healthy cell, a dying cell or a swelling cell. In addition, this new technique is easily adaptable to any microscope set-up, is fluorescent-label free, and is compatible with simultaneous fluorescence and/or other optical imaging and spectroscopy methods as well as magnetic separation and enrichment techniques. Other methods used to track morphological changes of single biological cells include Atomic Force Microscopy [21] (AFM) and Optical Tweezers [22] (OT). These methods may offer higher resolution, but are limited by the attachment of cells to a surface (AFM), or by the irreversible damage caused by laser trapping (OT). Furthermore with OT, for each cell line, viability studies have to be done for each cell type in order to prevent photodamage, which limits its applicability [23]. The use of cantilevers has also been reported to track the mass of live cells [24], but there are no publications yet on single cancer cells in suspension.

## Results

### Model for the rotation of magnetically labeled cells

**Figure 1:**
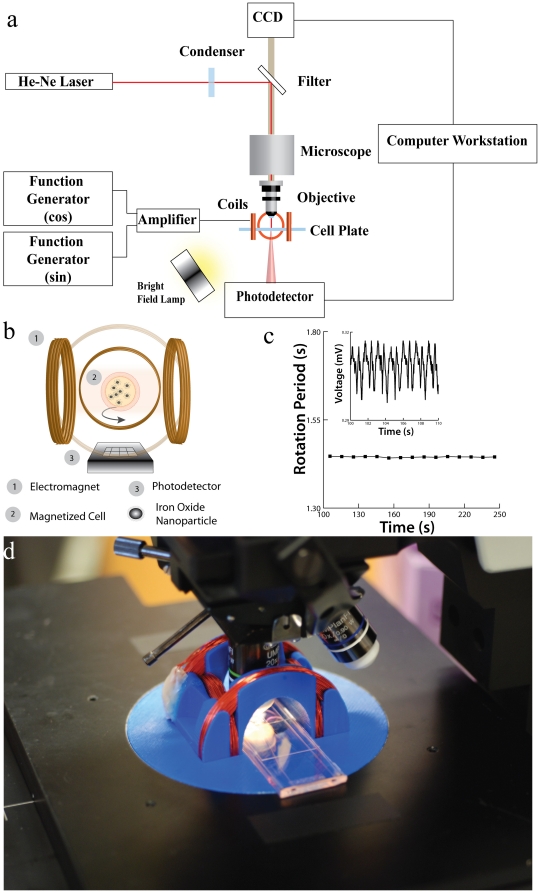
Magneto-Rotation of a single cell. **a**) Schematics of the complete setup. A Live Cell Array® plate, with 100 µm wells, is placed on the platform of a microscope, for which a set of electromagnets has been adapted. Note that the cell is not stuck to the bottom of the well. Under the 60× objective, the laser beam undergoes forward scattering from the rotating cell (15 to 20 µm), and the variations in the forward scattered light is captured in real-time by a photo-detector, and analyzed on a computer. **b**) Schematics of a rotating cell placed inside the magnetic coils: two identical sinusoidal signals, with a phase shift of 90°, pass through the two pairs of coils. The applied magnetic field and the magnetic moment of the cell are not aligned, creating a torque that drives the cell's rotation. **c**) Rotational period of a fixated cell in DMEM. The inset represents the raw signal from the photodetector, showing the periodicity over a given time window. The treatment of the signal then gives the rotational period (See **Methods** section on the optical setup for the signal treatment description). **d**) Caption of the setup. Custom Helmoltz Coils with NUNC Live Cell Array Plate on the microscope stage.

To verify that cells could be magnetically manipulated, we placed them in the center of magnetic coils with magnetic field amplitudes of 1 mT, as shown in **Figure 1b**. The coils themselves are adapted to the platform of a microscope in order to record videos (see **Supplementary Figure S4** and **Supplementary Video S1**). The single cells rotate at frequencies ranging from 0.05 Hz to 2 Hz in this setup (much lower than the 100 Hz driving fields, due to operating in the *asynchronous regime*, see below). Focusing a low power, 1.45 mW HeNe laser through the microscope, the forward scattered signal is recorded with a photodiode [25]. The cell viability is not affected by this low-intensity laser, as shown in **supplementary figure S3** and **supplementary tables S1** and **S2**. When the cell rotates, it produces rotational-dependent modulation that can be measured with the photodiode. With real-time signal processing, the rotation period of the cell and therefore its size/morphology can be monitored in real-time.

The cell is found to exhibit magnetic rotational behavior very similar to that of a magnetic microparticle (**Supplementary Fig. S1**). As shown by McNaughton et al. [26], and extended to the case of superparamagnetic particles [27], there exists a critical frequency of the external magnetic field above which the particle does not rotate synchronously with the field, i.e. the particle cannot keep up anymore with the driving frequency. In this asynchronous regime, the mean value of the rotation speed of the single cell is given by 
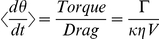
, where 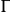 is the magnetic torque and 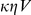 is the drag due to viscosity forces. Here, *κ* is its Einstein's shape factor, *V* the volume and *η* the coefficient of viscosity. We note that 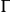 is proportional to the magnitude of the magnetic field, the magnetic moment of the cell and the volume of the magnetic *contents* of the cell; however, all these parameters are kept constant in the experiments. Therefore, in the asynchronous regime, any change in the cell's shape or volume, i.e. in its effective volume, 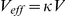, induces a change in the rotation speed, given by the above formula. This model has been further refined for the case of paramagnetic particles [28], [29], wherein the rotational period, 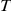, is found to be proportional to the effective volume, 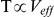 (this is true in the asynchronous rotational regime; for a complete derivation, see ref. 27 and equations in **Supplementary Information S1**). As can be seen from this dependence, if the volume increases, the rotation period increases proportionally. The same goes for the shape factor, and, as a consequence, one can detect morphology changes.

### Magnetic characterization of the cells

**Figure 2:**
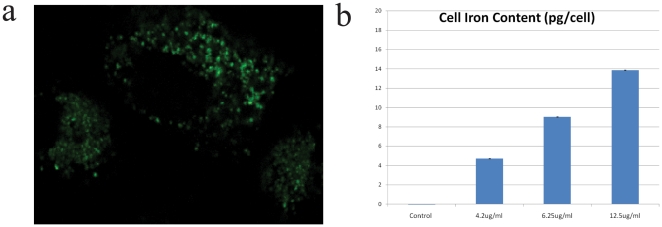
Magnetic content and nanoparticles localization of labeled HeLa cells. a) Fluorescence Image (40×) of a HeLa cells after incubation with dyed magnetic nanoparticles at an extracellular iron concentration of [Fe] = 12.5 ug/ml (0.22 mM). b) Cellular iron content in picogram per cell. The concentrations of particles in the media are given in iron concentration (error bars values represent mean +/−0.5*s.d., n = 3).

To characterize furthermore the magnetization of the cells, we looked at the localization of the nanoparticles after incubation, to determine whether they stayed attached to the surface, got internalized, and, if they did, if the nanoparticles were free to move in the cytoplasm or trapped in vesicles (endosomes). To do so, we attached HPTS fluorescent dyemolecules (8 - Hydroxypyrene - 1,3,6 - trisulfonic acid, trisodium salt) to our nanoparticles, using the electrostatic attraction forces between the particles and the dyes. HPTS is a membrane impermeant dye, and thus it needs a vector to get internalized by the cells. Following the standard protocol of incubation, we washed the cells three times in PBS, and the cells were observed under excitation at 450 nm with fluorescence being checked at 510 nm. The results are shown **figure 2a**. As we can see, the magnetic nanoparticles are internalized by the cell through endocytosis. In addition, neither the nucleus nor the cytoplasm shows fluorescence, which indicates that the nanoparticles remain in the vesicles. Moreover, we assessed the iron content of the cells by Inductively Coupled Plasma (ICP) measurement (see **Methods** section). As expected, the iron content increases with the MNP concentration in the culture media, and the trend appears to be linear in the concentration window that we used (**figure 2b**). For our rotation experiments, we estimated that the iron content is around 14 pg/cell. Compared to the mass of a nanoparticle, this means that, on average, less than 20,000 nanoparticles have gotten into the cell. Other sizes of magnetic nanoparticles were also tested (10 nm, 100 nm and 200 nm), but internalization was maximized for particles with a diameter of 30 nm (data not shown).

### Cytotoxicity assay and drug sensitivity

**Figure 3:**
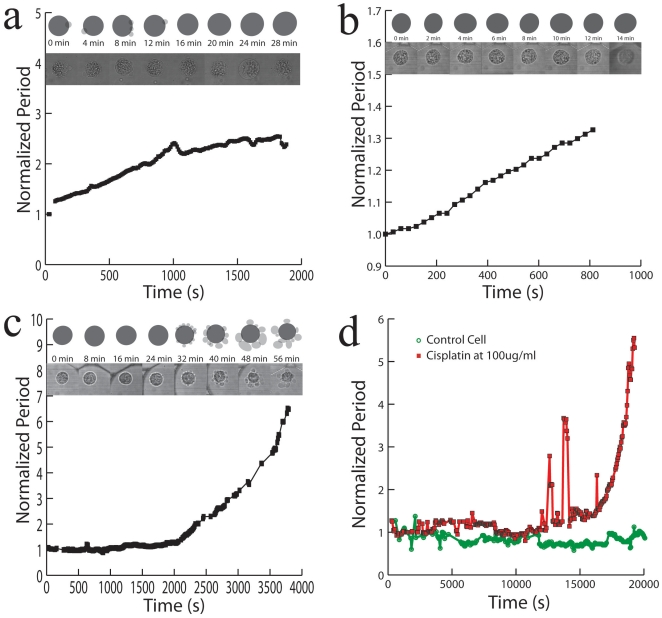
Changes in the rotation period of a single HeLa cell. **a**) In DMEM on an agarose layer **b**) In a mixture of 75% DI water and 25% DMEM **c**) In a mixture of DMEM with 5% Ethanol and **d**) for a live cell in DMEM (green circles) compared to a HeLa cell (red squares) in DMEM with a 100 ug/ml of Cisplatin. The Y axis is the normalized period, and the X axis is time in seconds. Lines show trend between connected points. For each graph, in the pictures above it, the bottom pictures show snapshots of the rotated cell at each indicated time, while the schematic pictures on top of it show the corresponding cell shapes (fixated cell not shown). Dark discs represent the cell cytoplasm and membrane, while grey spots show the vesicles formed at the surface, if any.

In this study, cancer cells loaded with nanoparticles were magnetically separated and resuspended in different media, such as culture medium (DMEM), DMEM with 5% Ethanol, DMEM with 100 ug/ml Cisplatin or DMEM with 75% deionifzed water. Each medium was used to verify different aspects of this method: DMEM was used as a control, ethanol was used as a cytotoxic agent, Cisplatin was used to model a drug assay; also, to promote stress through cell swelling, we used a large proportion of DI water, reversing the ionic balance between the inside and outside of the cell. Note that a large concentration of salt in solution has the opposite effect on the cell, namely shrinking it. The cells in suspension were then pipetted onto a Live Cell Array™ plate (NUNC™), where the array has 100 µm wide wells, which provide adequate compartments for single cells to rotate and be analyzed. Optical scattering signals (from the rotating cells) were recorded and the changes in the rotation period were measured for the different media (**figure 3**). Magneto-rotation was performed under numerous conditions, with different cell samples; the following results show typical examples of cell behavior that have been reproduced multiple times in our system.

**Figure 4:**
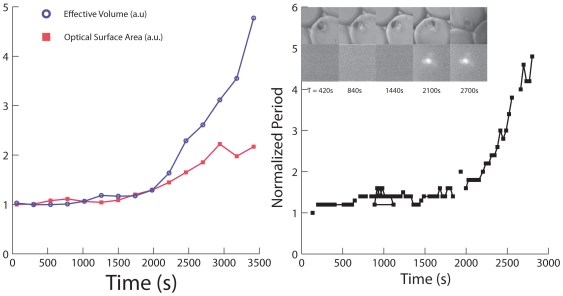
Sensitivity of the cell magneto-rotation method. **a**) Comparison of sensitivities between microscope and magneto-rotation in measuring cell death (HeLa cell in DMEM, 5% Ethanol). In red is the normalized surface area as measured with the microscope, and in blue is the normalized effective volume period as measured with Magneto-Rotation using **Supplementary Information S1**. **b**) Comparison of cell death monitoring using magneto-rotation and Live/Dead cell assay.

**Figures 3a and 3b** show two cases of cell swelling. Cell swelling generally occurs because of the osmotic pressure created either by an ionic imbalance, as mentioned earlier, or by a lack of nutrients. Either way, the cell expands to cope with the imbalance of the chemicals it needs for maintaining its metabolism. To reach ionic disparity, we used DI water (**figure 3b**). We also observed that cells would also swell when placed on an agarose layer (2% agarose in DI water) (**figure 3b**). Agarose gel is porous, a property that is used in the electrophoresis of proteins, and this property might be at the origin of the swelling. Indeed, the nutrients present in the growth media, mainly glucose, can diffuse into the agarose gel while the cells rotate above it. The cells would therefore swell to balance the reduced concentration of nutrients available in solution, as observed by Goldberg et al. in cortical cells [29]. Since the cell volume increases, the rotation period increases. Alternatively, cell death is provoked when placed in a solution with 5% ethanol (**figure 3c**) or using a concentration of 100 ug/ml of Cisplatin in solution (**figure 3d**, red line connecting squared dots). However, the mechanisms of these kinds of cell deaths are different from the cases above, since blebs appear at the surface of the cell. In 5% ethanol, it takes only around 30 minutes (**figure 3c**) for blebs to appear, while in the case of the treatment by Cisplatin at 100 ug/ml, it takes several hours. Contrary to the swelling case, it is the changes in shape of the cell membrane that increase the effective volume. Blebbing and the formation of vesicles at the surface of the cell indicate that the cell contents are being broken down and separated into several vesicles. As the death process continues, the vesicle sizes increase. This kind of phenomenon does not only add to the volume, but it critically affects the shape factor of the cell. The combination of these two parameters, namely the *effective volume*, is what is tracked with magnetorotation, thus amplifying the blebbing effect. Eventually, the drag on the cell becomes so high, compared to the initial state of the cell, that the cell rotation period rises drastically (by 550%), in a non-linear way (see **figures 3c**, red line on **figure 3d** and see **fig. 4** for a comparison with microscope measurements). Thus both cell death mechanisms, though very different, can be observed and differentiated with Cell Magnetorotation.

We also performed magnetorotation of a healthy cell (**figure 3d** **, green line**), in growth media. In the absence of a toxic agent, the rotation period did not significantly change (the standard deviation of the rotation period was 15%). A fixed morphology control test was realized by fixating the cells in a 4% formaldehyde vial (1.5 ml) for 10 min, under end-over-end vial rotation (see **supplementary figure S2, red line**). Since the membrane and the cell contents were cross-linked, the cell morphology did not change, under isotonic conditions, and thus, as expected, the rotation period did not change. As compared to a fixated cell, where the rotational period is very flat, for live cells we observe that the rotation period, over time, exhibits significant short-time fluctuations. This may be a result of the cell metabolism, which is still active during rotation. Overall, this shows that when the rotation period is constant, it corresponds to a cell that is not significantly changing in its effective volume.

To assess the accuracy of the method regarding effective volume modifications, we compared the trends in the effective volume (proportional to the rotation period) with those of the surface area, as estimated from microscopy images (the surface area being a standard indicator of the cell morphology/shape factor). With an imaging software (Adobe® Photoshop®), we estimated the surface areas of the cells at regularly spaced intervals. As can be seen on **figure 4a**, magneto-rotation is as effective as an optical microscopy setup for observing small changes. However, for bigger changes, magneto-rotation amplifies the response, compared to the optical microscope setup. Any significant loss of magnetic content takes several days, according to Arbab et al. [30]. Thus its impact on the interpretation of the results, after several hours, can be ignored. Also, the steady rotation rate of a magnetized control cell tends to confirm that the loss of magnetic content is not significant over the time-span of the measurement. Otherwise, the magnetic moment of the cell would critically decrease, and the cell would slow down significantly, which is not the case (**figure 3d**). Therefore, we can safely assume that the cell's effective volume is indeed proportional to the rotational period. Magneto-rotation can also be compared to Live/Dead cell assays. Cells were prepared following the protocol described earlier. Before pipetting into the microwell plate, we added 2.0 ul of calcein and 5.0 ul of propidium iodide (PI) to a 1 ml sample containing cells. Cells were left sit in the incubator for 10 min, and then resuspended in DMEM with 5% ethanol and the same amount of dyes, after which, they were pipetted and rotated. As can be seen on **figure 4b**, the cell undergoes morphology changes well before PI fluorescence can be seen, and by the time cell death (PI defined) occurs, the rotation period has slowed down by a factor of about 2. This not only shows that the magneto-rotation method' compares well with fluorescent assays, but also shows it to be more sensitive. It is not surprising to see the rotation rate slowing down *well before* one is being able to detect fluorescence from the PI dyes. Indeed, the PI dyes only make their way into the cytoplasm after the cell walls are have been destroyed. However, well before, other processes take place, one of them being the formation of blebs at the surface of the cell, a phenomenon that cell magneto-rotation can accurately monitor, which is not the case for the MTT assay for instance.

### Effects of magneto-rotation on cell viability and division

To investigate the ability of the setup to monitor cell death, without causing cell death, we conducted several viability tests (laser exposure, short term and long term effects of rotation on viability, cell division and clonogenicity).

**Figure 5:**
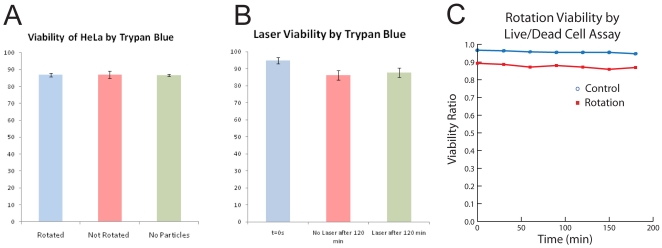
Magneto-rotation is harmless to the cells. **a**) HeLa cells viability after incubation with nanoparticles and rotation under a rotating magnetic field. All the cells came from the same cell line, and were cultured at the same time, each for 4 days. HeLa cells were grown until reaching 70% confluency, and the first sample constituted the control group (RHS). The two other groups, incubated with magnetic nanoparticles, originated from the same cell batch, cells grown in the presence of 40 ug/ml in DMEM, until reaching 70% confluency. Each group was made of two samples containing 50,000 cells each. While the second sample was not rotated, the third one (control) was put under a field of 0.5 mT and rotated at a driving frequency of 100 Hz (LHS). During the experiment, cells were maintained at 37°C, with 5% CO_2_ and humidity control. For every group, n = 3. Values represent mean +/−s.d. **b**) Magnetic HeLa cells viability before and after laser exposure. HeLa cells were incubated with magnetic nanoparticles, for 48 hours, following the protocol described before. In a 96-well plate, 150 ul of each set of cells was pipetted. Control measurement (blue) was realized after cells were washed, detached and resuspended in fresh media at 37°C. Non-exposed (red) and exposed cells (green) were kept on the microscope stage for 120 min at room temperature. Each well contained 25,000 cells. Values represent mean +/−0.5* s.d. n = 3. **c**) HeLa cells viability during magneto-rotation at 37°C, with humidity and 5% CO2 control. HeLa cells were pipetted onto a Live Cell Array (NUNC™). The cells trapped in the 100 um wells were counted using Calcein. For both the control and the rotated cells groups, n = 4. Cell death was monitored using Propidium Iodide. Standard deviations are within the dots.

We first tested the effect of the uptake of magnetic nanoparticles [yellow (RHS) and red (middle) bars in **figure 5a**], and of the presence of a magnetic field, on cell viability [red (middle) and blue (LHS) bars in **figure 5a**]. We performed the viability test on three different HeLa cell populations. After an hour at 37°C, with humidity and CO_2_ control, a cell count was made using Trypan blue. There was no significant difference in viability among the three cell groups (**figure 5a**). This shows that neither the incorporation of the particles nor the rotation under a magnetic field affected the cells viability over the time scale of an hour. Indeed, the same kind of magnetic iron oxide nanoparticles are quite commonly used [16], [30] to magnetophoretically separate certain cell populations from heterogeneous populations, as well as during MRI scans on patients (for contrast enhancement), without causing harm to cells. In the above viability tests, the field intensity and the magnetic particle concentrations were purposely set at higher values (0.5 mT and 40 ug/ml) than those described in this paper for magnetorotation (0.1 mT and 25 ug/ml), in order to keep a safety margin in the protocol.

Another possible concern we addressed is the effect of the laser exposure on the cell's viability (**figure 5b**). The viability test shows no significant cell death and no significant difference after two hours, between control cells and magnetic cells that were exposed to the laser. Both the interaction of the cells with light and the possible interaction of the magnetic nanoparticles with the laser do not affect the viability of the cells.

Finally, we investigated the possible impact of the physical rotation of the cells on their viability. Indeed, in order to accurately monitor toxicity effects, cell rotation has to be harmless. **Figure 5c** adresses this latter point. Comparing the death rate of rotating cells and the death rate of non-magnetic cells, we found no statistical difference in the two trends (n = 4, p = 0.245>0.05, F = 1.65<5.98 = F_crit_). In addition, as we observed (data not shown) and as described in other publications [30], cells containing magnetic nanoparticles can be subcultured. Also, to assess the cells' clonogenicity, we performed a clonogenic assay where cells were first magnetically rotated for 24 hours in an incubator, and then let to grow on agarose for three weeks. We found no significant difference between the control samples and the rotated samples (n = 3, t = 1.37<2.77 = t_crit_, p = 0.24>0.05 = p_crit_, see **supplementary figure S3**).

Finally, we also tested the effect of magneto-rotation on cellular division. The question was: does magneto-rotation impede immediate cell division? To investigate the short term impact, we rotated cells on agarose for 72 hours, and compared cell growth with two other controls (non labeled and magnetically labeled cells in the absence of magnetic field). We found no difference between the two different groups of magnetically labeled cells (see **supplementary figure S4**). This also rules out any potential magnetic hyperthermia happening during rotation.

## Discussion

### Harmlessness of the method

The use of magnetic nanoparticles and alternating magnetic fields has been commonly associated with hyperthermia, a process where the vibrating nanoparticles inside the cells produce heat, eventually killing the labeled cells through a rise in temperature. As a consequence, the ability to rotate cells through the internalization of similar magnetic nanoparticles and the application of a rotating magnetic field, i.e. alternating in two directions at the same time, without causing harm to the cell has been a concern, even though we are using much lower fields by an order of magnitude, and frequencies in the ranges of a few dozen Hz instead of a few 100 kHz [31], [32].

Our first concern was then to assure that the rotation in itself did not kill the cells. Our results show that the viability of the cells is preserved while they are rotated. Also the exposure to a (weak) laser (in order to capture a scattering signal from the rotating cell) does not have any effect on short term cell viability, as shown in **figure 5b**. However, the presence of a laser is not necessary, and the signal can also be analyzed through a camera, removing any long time risk that a long term exposure to a laser beam could cause.

Our results also show that the internalization of magnetic nanoparticles does not cause any effect on cell viability, and it only affects cell division by reducing the growth rate for a short time, over a limited number - at most 3 - of cell cycles, before reaching normal rates. Indeed, our magnetically labeled cells have been successfully subcultured in petri dishes, and we observed no difference in viability (see **Supplementary Information S1**) or in proliferation rates after three division cycles (data not shown). In accordance with previously published data [33], we also found that magnetically labeled cells grew at a slower rate than non-labeled cells, up until three division cycles, from which point onwards the growth rates were back to normal (see **Supplementary Information S1**). Also, as mentioned, according to Arbab et al. [30] the presence of cell internalized magnetic nanoparticles does not cause deleterious long term effects on the viability of the cells (over a period of 5 to 7 division cycles, i.e. over several weeks).

The presence of a rotating magnetic field, and the induced sub-hertz frequency rotations that were induced in the magnetically labeled cells did not have a long term impact on cell division, as shown by our clonogenic assay and by the cell count, after rotating cells for 24 to 72 hours.

Therefore, we have shown that for magnetorotation any cell death observed was the consequence of a purposely-induced toxic environment. In addition, we anticipate that since cells do not die as a result of rotation, cell growth, and even critical dormancy studies could be performed (work in progress). It is worth noting that cell division has been observed during rotation (see **supplementary video S3**), and rotating cells do not seem to have a different division rate compared to magnetically labeled non-rotating cells (see **Supplementary Information S1**) All in all, the difference in growth rate observed during rotation can be definitely associated with the labeling of the cells with nanoparticles, and not the impact of rotation itself.

This study presents a major difference in cell viability compared, for instance, to the *cell electro-rotation* method, which uses the cytolplasm non-uniformity to induce an electric dipole. The latter, at low frequencies, can cause the rupture of the plasma membrane, resulting in cell death [34].

In conclusion, cell magneto-rotation preserves the viability of the cell, both on a short and long term perspective (3 weeks). The rotation in itself does not affect cell growth. Our results hence demonstrate that if cells are harmed while they are rotated, it is caused by a harmful change in the cells environment.

### Cell magneto-rotation method potential relevance as a cytotoxicity and drug sensitivity assay

As described in the former section, we have demonstrated the ability to monitor cell death using the change in rotation rate of a magnetically labeled cell. The morphology of the cell has been successfully linked to cell fate, since we could associate the formation of blebs during cell death with a significant slowdown in rotation rate. We were also able to characterize cell death with a typical rotation trend, namely the exponential-shaped curve of the rotation period over time. Compared to a live/dead cell assay, we can detect cell slowdown as early as with fluorescence methods, if not earlier. Indeed, blebs are formed while the cell is dying, at a point where the cell membrane is still impermeable to the fluorescent dyes (here, propidium iodide). These results not only show the ability to discriminate cell death from the rotation curve shape, but also the compatibility of the method with a fluorescence assay. To this end, cell magneto-rotation can also be used as a way to maintain single cells in a non-adherent and localized fashion.

Another advantage of the presented method is its ability to track the very same cells over extended period of times. Indeed, fluorescent dyes are subject to photobleaching, affecting the evanescence of the intensity of the light emitted by the dyes. In order to monitor a phenomenon over time, it is then necessary to use different groups of cells that will be stained at different points of time.

As much as cell-to-cell variation can be screened by variations in fluorescence intensity in a cell sample, variations in the trends of cells' rotational periods can also give insights into cell-to-cell variability/heterogeneity. For instance, we can track this heterogeneity not only through the amount of iron-nanoparticles loading into the cell, but also through the time it takes for the rotational period to double under toxic conditions, in a similar fashion to the way the radiation half-life is measured for radio-active atoms. This way, the average «doubling time» will give a frame of reference for the entire cell population, while its distribution among cells in the same population will be a source of information regarding its heterogeneity.

Though we only show here single cells being studied, either separately or at a small throughput (between five to ten at the same time, see **supplementary video S3**), this study still serves as a proof-of-concept for the method, and our future work will be focused on more robust and perfected multiplexed arrays, with at least a few dozen cells, which would be the relevant quantity regarding circulating tumor cells. Cell magneto-rotation, rather than competing with techniques such as flow cytometry, complements them by extending the reach of the assays to rare cell populations that are naturally found in suspension, and by preserving them in this state while performing the assay.

In this study, our intention was to show that magneto-rotation could potentially be used as a novel method to monitor morphology changes of circulating tumor cells (CTCs)in suspension, at the single cell level. These cells are both very rare and, as stated by their name, are in suspension. They can even circulate in the bloodstream for months or longer [32] without attaching to any surface. This phenomenon, coupled with dormancy and repopulating potential, explains why patients who seemed apparently cured had developed one or several new tumors. In terms of adaptability, this new method can equally be used in serum (see **Supplementary Video S2**).

We have here introduced a new method to monitor morphology changes occurring in single cells in suspension. By keeping the cells in suspension, magneto-rotation could help bridge the gap between petri dish and bloodstream environment. Even though flow is not present in our system, the magnitude of the shear stress acting on the cell while rotating, is of the same order of magnitude as that in the bloodstream (20 to 40 dynes/cm^2^). It has to be noted though that shear stress in the bloodstream is not uniformly distributed in space and in time (due to heart pulses). Instead of a moving environment, the cell itself performs a relative motion, the advantage being that the cell stays highly localized, without the need to be attached or constrained, which would be the case if we wanted to track single cells in a flowing stream. In addition, it has been shown that gene expression and cell signaling are significantly different for cells grown on a 2D pétri dish compared to those grown in 3D [5], [6]. Once plated, clinical samples might also express a different phenotype than their suspended counterparts, a phenomenon that could be studied using Cell Magneto-Rotation. In the meantime, traditional assays, such as flow cytometry and MTT assays, have been relying on mass numbers and plated cells.

Hence, we see their potential inadequacy when it comes to toxicity assays of CTCs: the impossibility to perform these assays on a reduced number of cells (a few dozens), and, more seriously, the risk of being irrelevant because of the difference in gene expressions, if not mutations, that occur if these circulating cells are plated. Applied to the rare CTCs, where every single cell could be a repopulating one, the one that we want to target, and thus one cannot afford to lose a significant amount of cells at every time point of the monitoring. Another important feature that these cells exhibit is dormancy. They can stop growing for prolonged periods of time [35]. What is the point of plating these cells if they are not supposed to grow? And if they do grow, what conclusions can be drawn from assays made on cells that have been denatured in the process? As much as it is vital to «eradicate all intratumoral subclones», as stated by Notta et al. [11], the next anti-cancer therapies will also have to eradicate all the subclones in the circulating cell population so as to prevent metastasis: such drug sensitivity tests could be performed using the CM method, as a complementary technique. In addition, the magneto-rotation test can be used coupled with a camera instead of a laser beam (or an LED), and thus does not necessitate a complex optical setup besides the microscope. Since a dormant cell is alive but does not grow, its rotation rate should not vary under non-toxic conditions, even after a period of time corresponding to a full cell cycle. Thus our approach could allow us to discriminate dormant cells from the general population.

We chose to work on HeLa cells because of their ability not only to survive but also to grow in suspension. As such, for this proof of concept, they served as a model for CTCs. Many questions are worth being asked then. Do circulating or disseminating tumor cells divide in the bloodstream? If yes, which cells tend to divide? Do they mutate? Compared to plated cells, how do they respond to drugs? This Cell Magneto-Rotation method could potentially offer researchers a valuable tool to answer such questions.

We also observed the formation of filopodia in healthy cells during rotation. Filopodia are spikes that are responsible for cell motility, migration and fixation to a substrate. However, because filopodia are oriented toward the outside of the cell, these morphology changes were sufficient to affect the rotation rate. It is not clear yet whether filopodia formation is a result of rotation or a process that would occur anyways to cells in suspension. However, filopodia, or other protrusions, might not be formed in cells while circulating, but it is very likely that they appear when these circulating cells try to attach to the endothelium in order to reach for tissues and/or secondary tumors [36]. As such, if magneto-rotation actually permits the formation of protrusions, it could add a tool to the research effort on cell adhesion. Again, to our knowledge, no other method could do that for cells in suspension, which is critical when it comes to cells invading new tissues from the bloodstream.

In summary, we have described a single live cell analysis system that can monitor cell morphology through the related effective volume changes, *in suspension*; it does not affect cell viability. Specifically, we have demonstrated the ability to use cells as rotating magnetic microplatforms, through the uptake of functionalized magnetic nanoparticles, and the ability to control and measure their rotation under near real-time conditions. Cell death, and the dying process can simply be monitored through changes in the cell's rotational period. This lends itself to rapid drug sensitivity testing on cancer cells, with no need for cell culturing. Potentially, it could be used for tests on the rare and fleeting (due to differentiation) cancer stem cells. While circulating, the dormancy of these cells could also be evaluated this way, via the observed stability of their rotation rate. The methodology used here is very general, and can be used with various cell types (tumor, stem cells, red blood cells), and in various media. Also, this micro-system could be operated on a range of supports (cell imaging plate, agarose layer, inverted droplet, PDMS micro-channel), and we anticipate that this magneto-rotation method can also be applied to the rotation of other systems, such as cell clusters or spheroids (work in progress). The CM method here described could be adapted to various biotechnology applications, e.g. drug discovery or testing, and to growth assays, all performed in a three dimensional environment. We also envision CM integration into an *in vivo* magnetic enrichment process, followed by *ex vivo* monitoring, for tailor-made therapies. Ongoing work is focused on live cell analysis, on cell growth and on studies on clinical samples.

## Materials and Methods

### Functionalization of magnetic nanoparticles

To magnetically label HeLa cells, 30 nm amine coated magnetic nanoparticles (Ocean Nanotech®) were functionalized using poly-L-lysine (PLL, Sigma Aldrich GmBH), a transfection agent that improves the internalization in cells [30]. A solution of 200 ug/ml of nanoparticles in Dublecco's Modified Eagle Media DMEM was mixed with 10 uL of PLL, and rotated end-over-end in a vial at room temperature for 1 hour. The particles solution was then filtered using a 0.2 um filter (Whatman® Nylon Filter Media) to remove any biological agents that could contaminate the sample. The filtered solution was immediately used.

### Cell culture and labeling

HeLa 229 cells (American Type Culture Collection) were cultured for four days in Dublecco's Modified Eagle Media (DMEM 11995, Invitrogen™), 10% Foetal Bovine Serum (FBS), 1% Penicillin–Streptomycin–Glutamine (PSG) and 25 ug/ml (prior to filtration) of functionalized magnetic nanoparticles (Ocean Nanotech). The growth medium was removed, and cells were washed once, using PBS, before adding Cell Detachment Buffer (Gibco™). This enzyme free buffer does not affect surface proteins during cell removal from the dish, and allows the nanoparticles which could have attached the surface of the cell to be retained. After 30 min of incubation in the detachment buffer, cells were washed with DMEM, and centrifugated (for the preparation of fixated cells, this step was replaced by magnetic separation in order to keep the cells from forming clusters). Cells were resuspended in fresh media.

### Nanoparticles preparation for fluorescent imaging

3 ml of magnetic nanoparticles (tagged with poly-L-lysine) at a concentration of 200 ug/ml in DMEM were mixed with 3 mg of HPTS fluorescent dyes. The mixture was vortexed and then put under end over end rotation for one hour before being centrifuged at 9000 rpm for ten minutes in Amicon® Ultra centrigugal filters Ultracel® 3 k. The particles tagged with the fluorescent dyes were then resuspended in DMEM at the initial concentration of 200 ug/ml.

### Setup for rotation measurements

Before rotation, 300 uL of the cell solution was introduced into a Live Cell Array™ plate (NUNC®), with 100 um wells. Cells were then pulled to the bottom of the plate using a permanent magnet. Once cells were pulled down to the wells, the plate was placed inside the coils, with the wells in the center.

### Coils description

Custom Helmholtz coils (see **figure S1**) were integrated on the platform of an Olympus® BX50WI microscope. Each pair of coils produced a field parallel to the imaging plane and was plugged into an amplifier (amplification factor during rotation was set to 1), which, in turn, was plugged to two function generators with a 90 degree phase shift (Agilent Technologies Arbitrary Waveform Generator 33220A, 20 MHz function). Both power supplies were set to provide a sine wave function, with amplitude of 3V. The phase shift was controlled with an oscilloscope (Agilent Technologies, DSO5012A). Finally, the magnitude of the magnetic field was measured using a magnetic probe placed in the center of the magnetic coils (3 Axis Magnetic Field Transducer, C-H3A-2m_E3D-2.5kHz-1%-2T, Sensitivity 5[V/T], SENIS GmbH).

### Optical setup

The laser used was an unstabilized HeNe laser (Spectra-Physics® 136/P), with a wavelength of 632 nm. Data were acquired using a Labjack UE9 data acquisition device, receiving the diffraction signals from a non amplified photosensor. The data were recorded analyzed on a computer (DELL©, *Intel® Core™2 Duo CPU E6550 at 2.33Ghz, 1.98GB RAM, Microsoft Windows® XP Professional Version 2002 SP3) using customized software (LabView).*

The modulated signal is then treated using an algorithm that recognizes the peak to peak variations. From there, the rotation period is extracted averaging the peak to peak period over a defined time window that moves over time. For instance, the time window over which we average the period could be 60 s, and it would be translated by twenty seconds to calculate the next point.

The longest cell rotation period used is on the order of one minute, which is the case when the cell's blebbing created a large cell and a high effective volume. At the beginning of the experiments, the rotation period was usually comprised between 1 s and 15 s. To analyze the signal, we measured and average the rotation period over a moving time window of at least 10 periods. In the early stages, we needed a 30 s time window, and when the rotation rate becomes very low (30 s), we used a time window of around 3 min (even though at this point, a statistical averaging of the rotation period is not relevant since the length of the period reduces the error made on the measure).

*Image acquisition was made through a Digital Camera (Mightex Monochrome Camera MCE-B013-US, 1.3 MegaPixels), and images were recorded with the Mightex acquisition software (v1.1.0, 1280x1024, Exposure Time 35 ms). Image capture was realized via an external trigger, programmed on LabView.*

Fluorescent imaging was performed on a Leica Inverted SP5X Confocal Microscope System with 2-Photon FLIM housed at the Microscopy and Image-analysis Laboratory (MIL) at the University of Michigan

### Laser wavelength, power

The laser power was measured using a power-meter (Coherent Calibration Tag, MIL-STD-45662-A). Before reaching the microscope's mirror (namely after its transmission through the condenser), the power measured was of 1.45 mW. On the microscope platform, the power was between 125 uW +/−2 uW.

### Inductively Coupled Plasma

After the standard incubation protocol, cells were washed three times in ice-cold PBS, detached, and counted. Afterward, cells were digested for three hours in 70% nitric acid in a water bath at 90°C and the iron content was then measured using an Inductively Coupled Plasma (ICP). For these measurements, the magnetic nanoparticles were not filtered before incubation, so that the exact density in solution was known. To make sure the MNP concentration was the same with unfiltered particles than with filtered ones, we measured the iron content of the MNP solution before and after filtration, resulting in a loss of 50% of the particles (data not shown).

## Supporting Information

Figure S1 **Frequency response of a fixated cell.** (error bars are inside the dots, values represent mean +/−0.5*s.d. , n = 18).
(TIF)

Figure S2 **Changes in the rotation period of a single HeLa cell.** In DMEM (blue circles) compared to a fixated HeLa cell (red squares) in DMEM.
(TIF)

Figure S3 **Clonogenic assay on HeLa cells.** HeLa cells incubated with magnetic nanoparticles (12.5 ug/ml, unfiltered) and rotated for 24 hrs in an incubator. For each sample, after incubation with magnetic nanoparticles following the standard protocol, cells were washed, detached and counted. 10000 cells were then rotated for 24 hrs at 37°C, in a 5% CO2 environment with humidity control. Using a 6-well plate, 200 cells were put to grow on an agarose layer (1.3% agarose in DMEM) for 3 weeks. Control cells were not exposed to nanoparticles nor to any magnetic field. Control cells were washed, detached, counted and for each well, 200 cells were put to grow on agarose. Values represent mean +/−0.5* s.d. n = 3.
(TIF)

Figure S4 **Effect of rotation on cell division.**
(TIF)

Table S1 **Magnetic HeLa cells viability before and after exposure.**
(DOC)

Table S2 **t-Test comparing non-exposed and exposed HeLa cells.** The mean of each variable is given as the fraction of viable cells (Two-Sample Assuming Unequal Variances).
(DOC)

Video S1 **Real time video of a magnetized HeLa cell.** HeLa cell in DMEM in a 100 um well, under a rotating magnetic field. Frame rate is 26 fps.
(AVI)

Video S2 **Video of a magnetized HeLa cell in goat serum.** (Goat Serum Donor Herd, G6767, Sigma-Aldrich®, USA) in a 100 um well, under a rotating magnetic field. Frame rate is 5 fps.
(AVI)

Video S3 **Video of a magnetized HeLa cell undergoing mitosis.** (in DMEM in a 100 um well, under a rotating magnetic field. Frame rate is 25 fps).
(AVI)
