# Supplementary figures and images for: Nanoparticle Induced Cell Magneto-Rotation: Monitoring Morphology, Stress and Drug Sensitivity of a Suspended Single Cancer Cell
Source: PLoS One. 2011 Dec 13;6(12):e28475. doi: 10.1371/journal.pone.0028475 (PMC3236752; doi:10.1371/journal.pone.0028475)

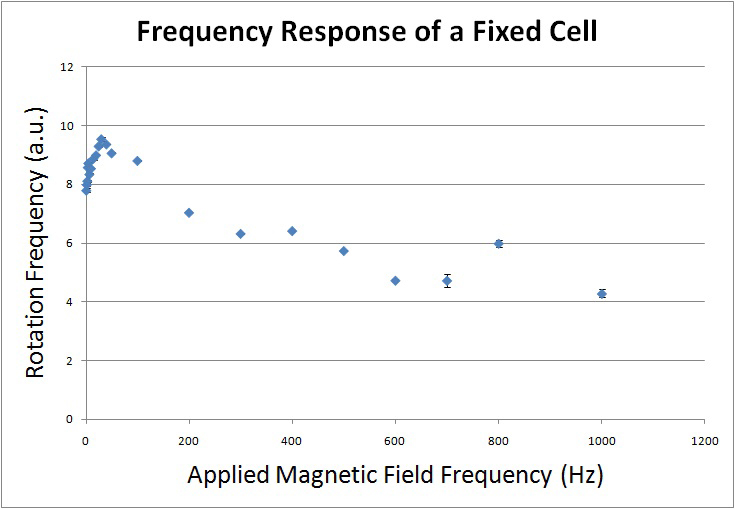

Supplement: Figure S1 — Frequency response of a fixated cell. (error bars are inside the dots, values represent mean +/−0.5*s.d. , n = 18). (TIF) [file pone.0028475.s001.tif]

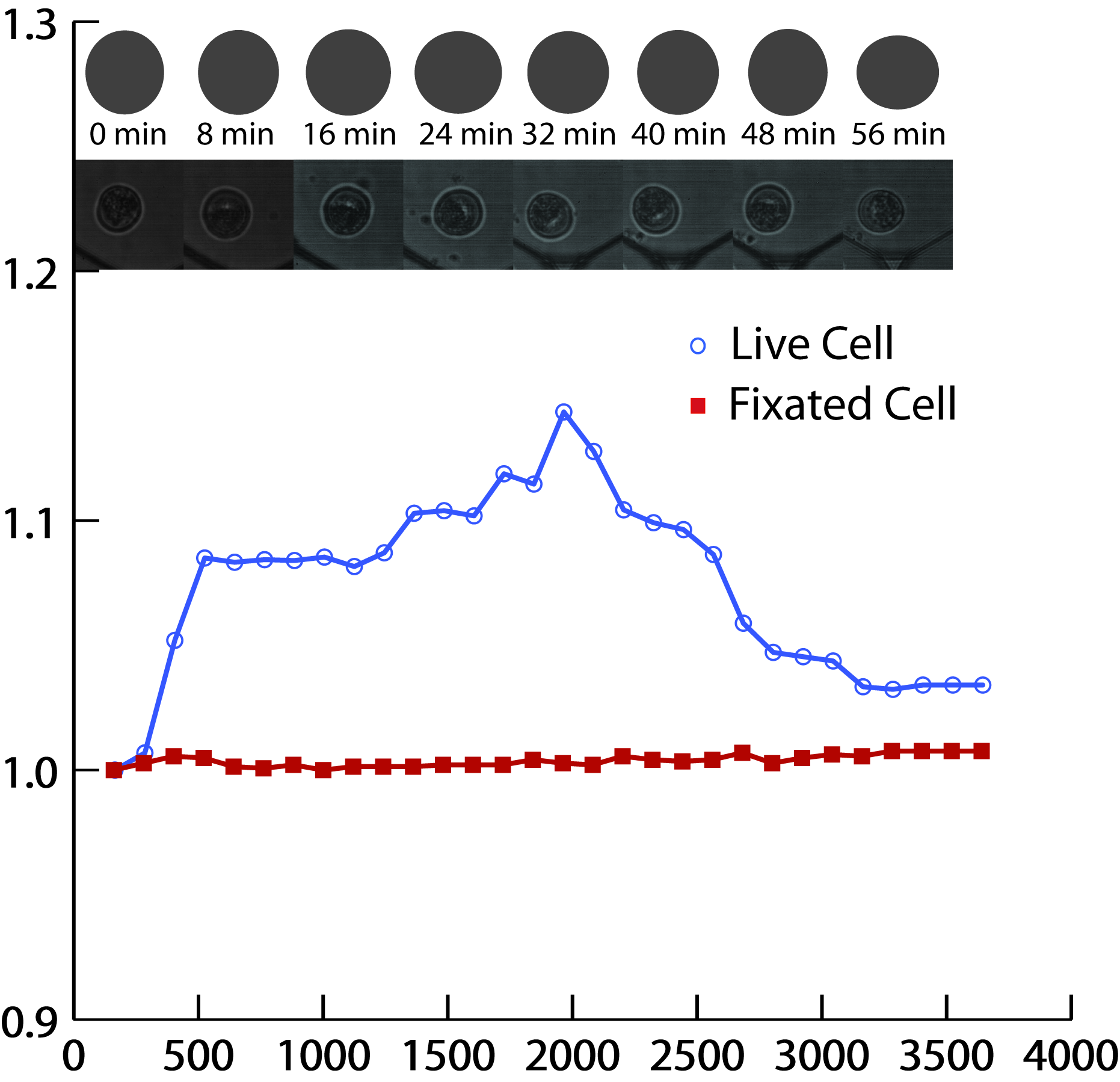

Supplement: Figure S2 — Changes in the rotation period of a single HeLa cell. In DMEM (blue circles) compared to a fixated HeLa cell (red squares) in DMEM. (TIF) [file pone.0028475.s002.tif]

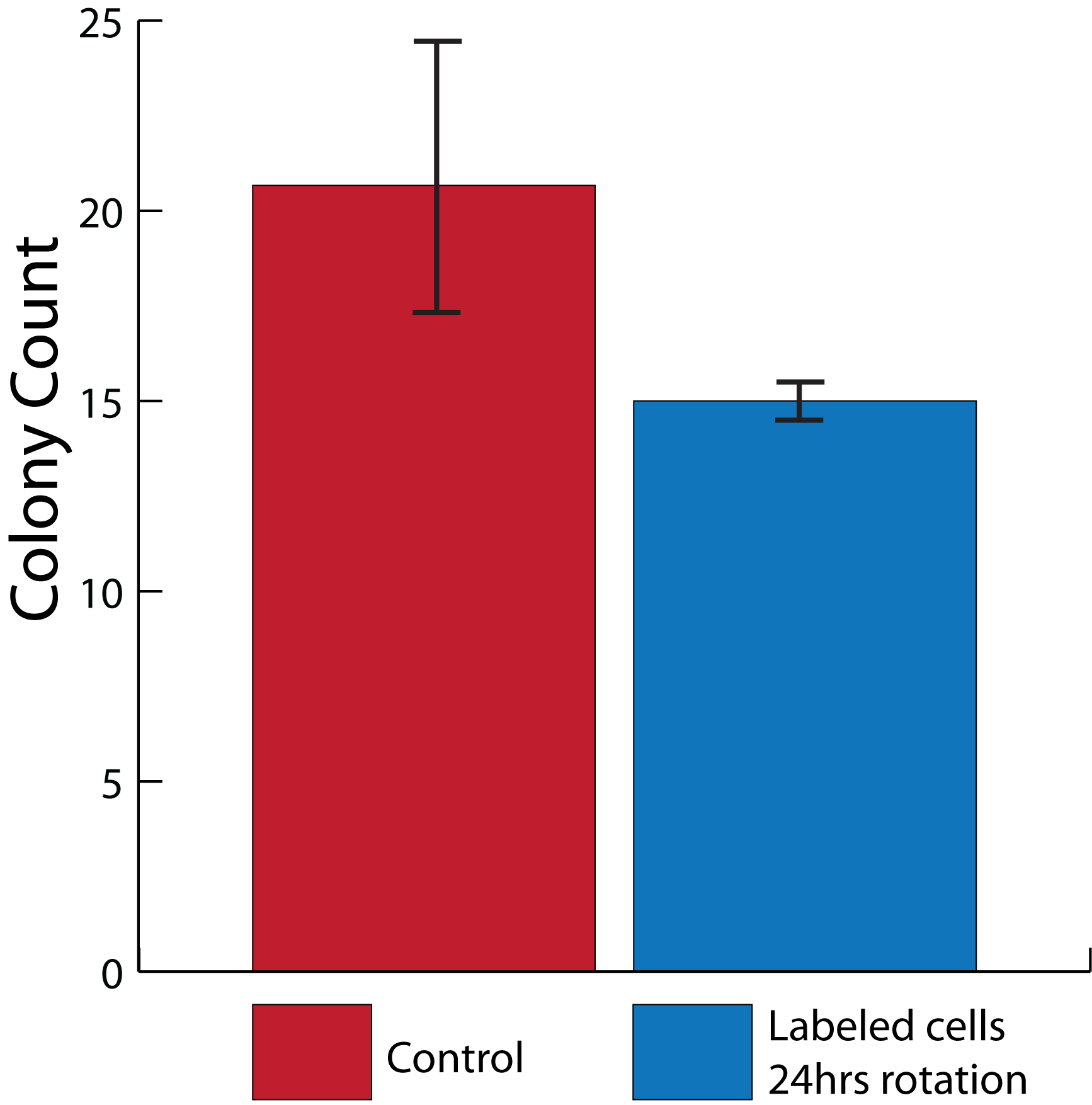

Supplement: Figure S3 — Clonogenic assay on HeLa cells. HeLa cells incubated with magnetic nanoparticles (12.5 ug/ml, unfiltered) and rotated for 24 hrs in an incubator. For each sample, after incubation with magnetic nanoparticles following the standard protocol, cells were washed, detached and counted. 10000 cells were then rotated for 24 hrs at 37°C, in a 5% CO2 environment with humidity control. Using a 6-well plate, 200 cells were put to grow on an agarose layer (1.3% agarose in DMEM) for 3 weeks. Control cells were not exposed to nanoparticles nor to any magnetic field. Control cells were washed, detached, counted and for each well, 200 cells were put to grow on agarose. Values represent mean +/−0.5* s.d. n = 3. (TIF) [file pone.0028475.s003.tif]

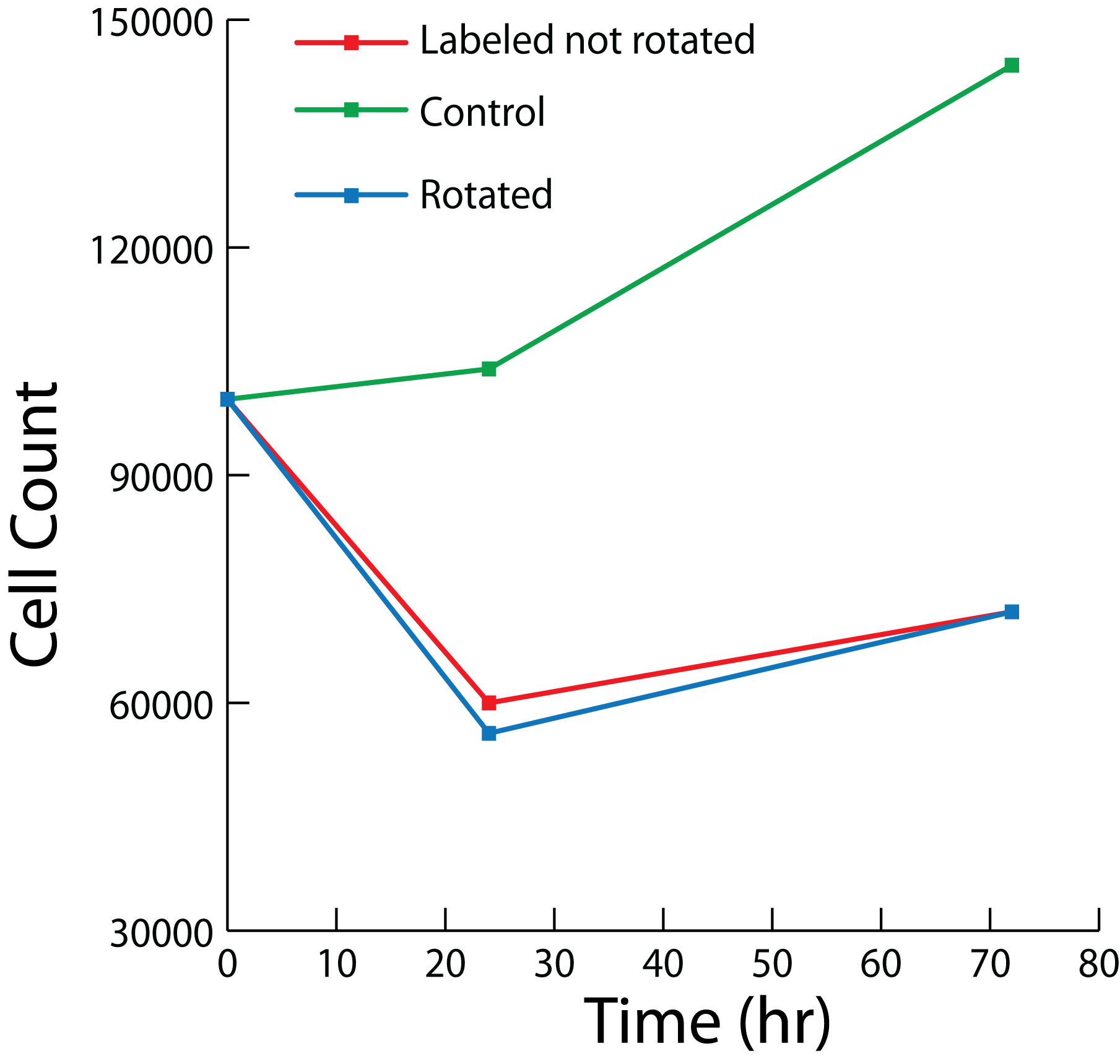

Supplement: Figure S4 — Effect of rotation on cell division. (TIF) [file pone.0028475.s004.tif]
