# Supplementary material for: Nanoparticle Induced Cell Magneto-Rotation: Monitoring Morphology, Stress and Drug Sensitivity of a Suspended Single Cancer Cell
Source: PLoS One. 2011 Dec 13;6(12):e28475. doi: 10.1371/journal.pone.0028475 (PMC3236752; doi:10.1371/journal.pone.0028475)
Supplement: Table S1 — Magnetic HeLa cells viability before and after exposure. (DOC) [file pone.0028475.s005.doc]

| Sample | T=0 (n=3) | | No Laser after 120 min (n=4) | | Laser after 120 min (n=4) |
| --- | --- | --- | --- | --- | --- |
| Viability | 94.848382 | | 86.24060558 | | 87.84646275 |
| Standard Deviation | 3.6802803 | | 5.418544922 | | 5.666348878 |
|  | | | |  | |
|  | |  | |  | |
