# Supplementary material for: Nanoparticle Induced Cell Magneto-Rotation: Monitoring Morphology, Stress and Drug Sensitivity of a Suspended Single Cancer Cell
Source: PLoS One. 2011 Dec 13;6(12):e28475. doi: 10.1371/journal.pone.0028475 (PMC3236752; doi:10.1371/journal.pone.0028475)
Supplement: Table S2 — t-Test comparing non-exposed and exposed HeLa cells. The mean of each variable is given as the fraction of viable cells (Two-Sample Assuming Unequal Variances). (DOC) [file pone.0028475.s006.doc]

|  | *Variable 1 (No Laser 120min)* | *Variable 2 (Laser 120min)* |
| --- | --- | --- |
| Mean | 0.862406056 | 0.878464627 |
| Variance | 0.002936063 | 0.003210751 |
| Observations | 4 | 4 |
| Hypothesized Mean Difference | 0 |  |
| df | 6 |  |
| t Stat | -0.409648981 |  |
| P(T<=t) one-tail | 0.348143532 |  |
| t Critical one-tail | 1.943180274 |  |
| P(T<=t) two-tail | 0.696287064 |  |
| t Critical two-tail | 2.446911846 |  |
